# Regulation of static and dynamic balance in healthy young adults: interactions between stance width and visual conditions

**DOI:** 10.3389/fbioe.2025.1538286

**Published:** 2025-01-28

**Authors:** Wei Luo, Zhirui Huang, Hao Li, Tao Zhong, Peishun Chen, Yu Min

**Affiliations:** Department of Rehabilitation Medicine, The Affiliated Panyu Central Hospital, Guangzhou Medical University, Guangzhou, Guangdong, China

**Keywords:** stance width, visual input, static balance, limits of stability, healthy adults

## Abstract

**Objectives:**

This study investigates the impact of five distinct stance widths on static balance and limits of stability in healthy adults under varying visual conditions, specifically with eyes open and closed.

**Methods:**

The Prokin Balance Instrument was used to evaluate static balance with both eyes open and closed, as well as limits of stability with eyes open, in 20 healthy adults (male, age = 21.55 ± 1.39). Participants were assessed at five stance widths (0 cm, 10 cm, 20 cm, 30 cm, and self-selected width) for each condition. Statistical analysis of the test indices was conducted using repeated measures ANOVA.

**Results:**

In static balance tests, index values were higher with eyes closed than with eyes open, with this difference being most pronounced at narrower stance widths. Notably, a significant main effect was observed for all indicators, in the static balance test under varying visual and stance width conditions (P < 0.001). Significant interactions between visual conditions and stance width were identified for all static balance indicators (P < 0.001), except for average speed of anteroposterior sway (P = 0.195). Across both visual conditions, static balance indicators displayed a U-shaped distribution (a decrease followed by an increase) with increasing stance width, reaching a minimum at the self-selected width (16.35 ± 4.20 cm) and 20 cm. Additionally, significant main effects were observed for limits of stability in both the anteroposterior and mediolateral directions (P < 0.001). The limits of stability in the anteroposterior and mediolateral directions increased monotonically with stance width, peaking at 30 cm. The influence of stance width on static balance and limits of stability was significantly greater in the mediolateral direction compared to the anteroposterior direction, regardless of visual condition.

**Conclusion:**

As stance width increases, the reliance on visual input for maintaining static balance decreases in healthy adults. We recommend using a self-selected stance width to optimize static balance and a 30 cm stance width to achieve maximum limits of stability during postural assessments.

## 1 Introduction

Balance refers to an individual’s ability to automatically adjust and maintain posture both at rest and in motion, as well as in response to external stimuli (Lopes et al., 2014). Human balance consists of static and dynamic components. Static balance is the ability to maintain stability in a fixed posture, such as sitting or standing, while dynamic balance involves the automatic adjustment and maintenance of posture during movement or in response to external forces. Dynamic balance is further divided into autonomous and passive dynamic balance. Autonomous dynamic balance refers to the capability to restore balance during self-initiated movements, such as transitioning between postures like standing and sitting. In contrast, passive dynamic balance refers to the ability to regain balance when faced with external disturbances, such as pushing or pulling (Goldie et al., 1989; Sell, 2012; Shumway-Cook and Woollacott, 2017). The primary distinction between static and dynamic balance is that static balance involves maintaining the body’s center of gravity in a specific position, whereas dynamic balance requires continuous control of the body’s center of gravity to enable precise and smooth movement execution (Bizzi et al., 1992).

Static balance and limits of stability are fundamental to human movement and daily activities (Marchesi et al., 2022; Shin et al., 2020). These capabilities not only impact athletic performance but are also crucial for safety and quality of life. With an increasing emphasis on health and sports science research in contemporary society, establishing precise postural tracing standards is essential. Such precision enhances the comparability of balance testing and training outcomes, enabling clinicians to accurately diagnose balance function and devise effective training programs (de la Torre et al., 2017).

As the global population ages, the incidence of falls is on the rise (Xu et al., 2022; Dominguez, 2020), underscoring the need for effective balance training strategies to improve balance and prevent falls. Therefore, a comprehensive understanding of the factors influencing balance function is vital for enhancing individual quality of life and promoting public health.

Balance function is influenced by various factors, including visual, vestibular, and proprioceptive inputs (Teaford et al., 2023; Koelewijn and Ijspeert, 2020), the type of insole or footwear (Błażkiewicz and Hadamus, 2024; Park et al., 2023), foot posture (Al Abdulwahab and Kachanathu, 2015; Ghorbani et al., 2023), standing posture (Kędziorek and Błażkiewicz, 2022; Riis et al., 2020), and muscle strength (Bugalska et al., 2022; Feng et al., 2024). Biomechanically, the primary factors influencing human balance include three critical dimensions: the height of the body’s center of gravity, the area of the support surface, and the stability of that surface (Pollock et al., 2000). Physiologically, human balance proficiency is closely linked to the functions of the visual, vestibular, and proprioceptive systems (Koelewijn and Ijspeert, 2020). Clearly, maintaining human balance relies on the synergistic interplay and cooperation of these multiple factors, and any disruption within these components can compromise the body’s balance regulation mechanism.

Previous studies have investigated the impact of stance width on balance function. It is well-documented that variations in stance width affect postural control and shooting stability, particularly noting a decrease in stability with increased stance width (30–90 cm heel-to-heel spacing). Thus, the recommendation of a wider stance for enhancing shooting performance warrants reconsideration, as a stance width of 30 cm may provide an optimal balance between postural stability and shooting performance (Hawkins and Sefton, 2011).

Furthermore, research on various stance positions has shown significantly greater balance stability when standing with feet parallel at shoulder width, compared to standing with feet together or at a 30-degree foot declination angle. This indicates that changes in bipedal stance positioning can influence the available base of support, thereby affecting the body’s center of mass relative to bipedal stance stability (Scoppa et al., 2017). However, there is ongoing debate about the ideal stance width for optimal postural control. Some researchers propose a 5 cm heel spacing, 30°toe abduction, or a 15 cm parallel position over a bipedal contact position (Scoppa et al., 2017). Interestingly, while self-selected foot positions have been recommended for exercise or postural testing, this approach lacks definitive validation in the literature (Kirby et al., 1987). Additionally, studies indicate that reducing the support area has a lesser impact on stability in healthy individuals but a more pronounced effect on those with impaired balance function, especially in the medial-lateral direction (Mehdikhani et al., 2014).

The findings from previous research clearly demonstrate the significant influence of stance width on balance control within a fixed plane. However, these studies have primarily concentrated on static standing balance, with limited investigation into how stance width affects dynamic balance posture. Consequently, a definitive recommendation regarding the optimal stance width for balance posture remains elusive (Schmidle et al., 2022). While some studies have investigated the impact of changes in support area on balance function, the lack of standardized standing postures across these studies complicates the assessment of how such changes specifically affect balance function.

Previous research has highlighted the crucial role of vision in both static and dynamic balance postures. Studies have shown that visual deprivation can increase body sway by more than one-third in healthy adults (Butler et al., 2008). Narrow single-legged stances are more susceptible to visual movement effects compared to wider two-legged stances (Asseman et al., 2005), with these effects being more pronounced in younger individuals than in older adults (Benjuya et al., 2004). While vision is known to compensate for deficits in other sensory modalities, some researchers argue that despite visual dependency, vision may not fully compensate for other sensory deficiencies (Rinaldi et al., 2009; Meier et al., 2016). Furthermore, evidence suggests that various parameters of visual response to body sway exhibit differential sensitivity to visual manipulation. Traditional parameters such as amplitude and velocity are particularly responsive to visual perturbations, especially in the context of narrowing and altering the shape of the stance substrate. In contrast, parameters like frequency and capture time appear less affected by visual changes (Sarabon et al., 2013).

Changes in stance width can alter the sensory inputs involved in balance control, leading to sensory reweighting. As stance width increases, active balance stabilization mechanisms may become more prominent, engaging muscles and potentially enhancing proprioceptive inputs, thereby reducing the reliance on visual cues for postural control (Schweigart and Mergner, 2008). However, a definitive recommendation on the optimal range of stance widths has yet to be established.

While the individual effects of vision and stance width on balance function have been well documented, the interaction between these factors remains relatively unexplored, potentially limiting the scope of the study’s conclusions. This aspect is significant from both clinical and research perspectives.

This study aims to systematically analyze the effects of stance width on static balance and limits of stability under varying visual input conditions. The findings will serve as a reference for developing standards and guidelines for postural tracing and are expected to provide practical guidance for exercise balance training and health management. Additionally, this research will offer a scientific foundation for fall prevention, which is particularly significant in the context of an aging society.

We hypothesize that in the static balance test, stability will exhibit a U-shaped distribution: initially increasing and then decreasing as stance width expands. Conversely, in the limits of stability test, we anticipate a monotonic increase in the maximum excursion with greater stance width. Additionally, we expect balance stability to be lower in the eyes-closed condition compared to the eyes-open condition. Furthermore, we propose an interaction between vision and stance width, hypothesizing that the reliance on vision for maintaining balance posture will gradually diminish as stance width increases.

## 2 Materials and methods

### 2.1 Participants

The sample size estimation was calculated using a significance level of 0.05, a power of 0.822, and an effect size set at 0.8. Moreover, existing research indicates that gender might influence postural control in humans, although this remains a topic of scientific controversy (Ozcan Kahraman et al., 2018). Therefore, twenty healthy male adults participated in this study. None of the participants were athletes or sports enthusiasts, and all had no history of motor or neurological disorders, dizziness, balance issues, lower extremity fractures, muscle tears, or surgeries. Additionally, none were taking medications that could affect the central nervous system or balance function. Participants abstained from alcohol for 7 days and coffee for 3 h prior to the official test. All subjects were right-side dominant. The study was approved by the Ethics Committee of Panyu Central Hospital, Guangzhou Medical University (PYRC-2021-078), and informed consent was obtained from all participants before the experiment. The trial was also registered on https://www.chictr.org.cn/(Registration No.: ChiCTR2400082130).

### 2.2 Test program

#### 2.2.1 Testing instruments and requirements

The Prokin Balance Model 252 (TECNOBODY, Italy) is an advanced postural technology device that includes key components such as a display, an elliptical balance pedal, and contact sensors. It provides precise quantitative assessment and training for both dynamic and static balance functions, tailored to the diverse training needs of patients through an adjustable damping function with 50 levels in four directions. Additionally, its ability to accurately measure platform angles (±15°) and torso sensor angles (±30°) ensures a high level of accuracy and reliability during assessments.Designed to meet international medical device standards, the Prokin Balance 252 can accommodate loads up to 150 kg with a load resolution of 100 g, making it suitable for a wide range of patient populations. Its user-friendly interface enhances its utility as an essential tool for evaluating and improving balance functions in the field of rehabilitation medicine. Previous studies have demonstrated that the Model 252 Prokin Balance is capable of detecting subtle differences or impairments that conventional clinical scales might overlook (Lin et al., 2020). This capability helps to mitigate the ceiling effect often associated with scale-based assessments. This critical attribute was a significant factor in our decision to select the Prokin Balance Instrument as the testing tool for this study.

**FIGURE 1:**
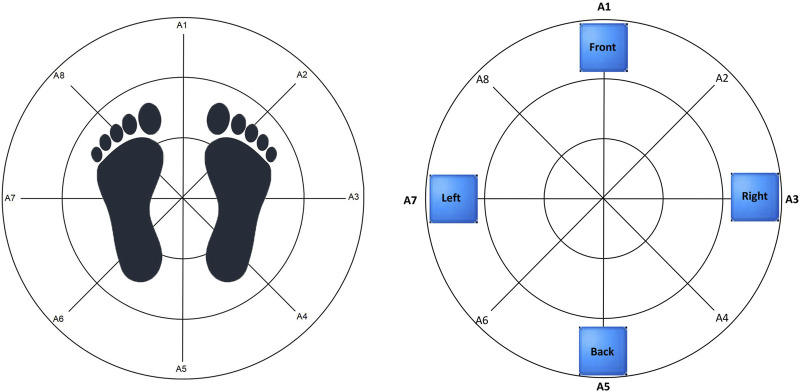
Presents a top-view schematic illustrating the standard standing position and the movement directions during the limits of stability test.

Before the test commenced, the researcher provided participants with a detailed explanation of the process, methodology, objectives, and specific posture requirements, ensuring their full understanding and cooperation with the testing protocol. To minimize potential external influences such as environmental conditions and clothing, participants were instructed to wear loose-fitting attire, perform one or two pre-test exercises, and maintain a quiet testing environment. These measures were implemented to ensure the accuracy and reliability of the data. During the test, subjects stood barefoot on the pressure platform of the balance instrument, maintaining a horizontal and upright posture with both feet, avoiding outward or inward toe positions. The inner ankles of both feet were aligned with the baseline, hands naturally hanging at their sides, and eyes focused on an achromatic target 1 m ahead. See Figure 1 for reference.

#### 2.2.2 Test composition

The balance assessment consisted of three components: open-eye static balance, closed-eye static balance, and open-eye limits of stability test. Participants were evaluated at five distinct standing widths (0 cm, 10 cm, 20 cm, 30 cm, and self-selected width). We employed a computer-generated random sequence to determine the order of precedence during testing.

The standing width refers to the distance between the centers of the inner boundaries of the two feet. To ensure consistency in measurements, the standing position was marked on the pressure platform. During the static balance test, participants were required to maintain a stationary position for 30 s, with each test repeated twice. In the limits of stability test, participants were instructed to shift their center of gravity to the furthest point in four directions (forward, backward, left, and right) quickly and accurately, as indicated on the computer screen, without moving their feet, until all movements were completed (See Figure 1 for reference.). This test was also repeated twice.

#### 2.2.3 Observation indicators

Based on the results of previous studies (Sarabon et al., 2013; Hébert-Losier and Murray, 2020; Thomsen et al., 2017), we selected the following highly reliable and sensitive parameters as the main observational indices for this study: in the static balance test, we recorded the participants’ average speed of anteroposterior sway (APAS), average speed of mediolateral sway (MLAS), area of body sway (SA), and length of body sway (SL) under both eyes-open and eyes-closed conditions. Larger values of these indicators suggest poorer static balance stability (Dumanlidağ and Milanlioğlu, 2021).

For the limits of stability test, we measured the deviation of the participants’ center of gravity in the four directions (front, back, left, and right) as a percentage of the preset deviation. A larger deviation amplitude, reflected in a higher percentage value, indicates better limits of stability function (Xu et al., 2021).

#### 2.2.4 Statistical analysis

Statistical analyses were performed using SPSS 21.0 software. Descriptive statistics are presented as mean ± standard deviation, and data normality was evaluated using the Shapiro-Wilk test. A two-way repeated measures analysis of variance (rmANOVA) was conducted to examine the differences in static standing balance across two conditions (stance width×vision). When interactions were significant, a simple effects analysis was performed. Additionally, a one-way repeated measures ANOVA was used to assess differences in limits of stability across varying stance widths. When the Mauchly’s sphericity test indicated a violation, we applied the Greenhouse-Geisser correction. Additionally, pairwise comparisons were performed using the Bonferroni adjustment for confidence intervals.

## 3 Results

**FIGURE 2:**
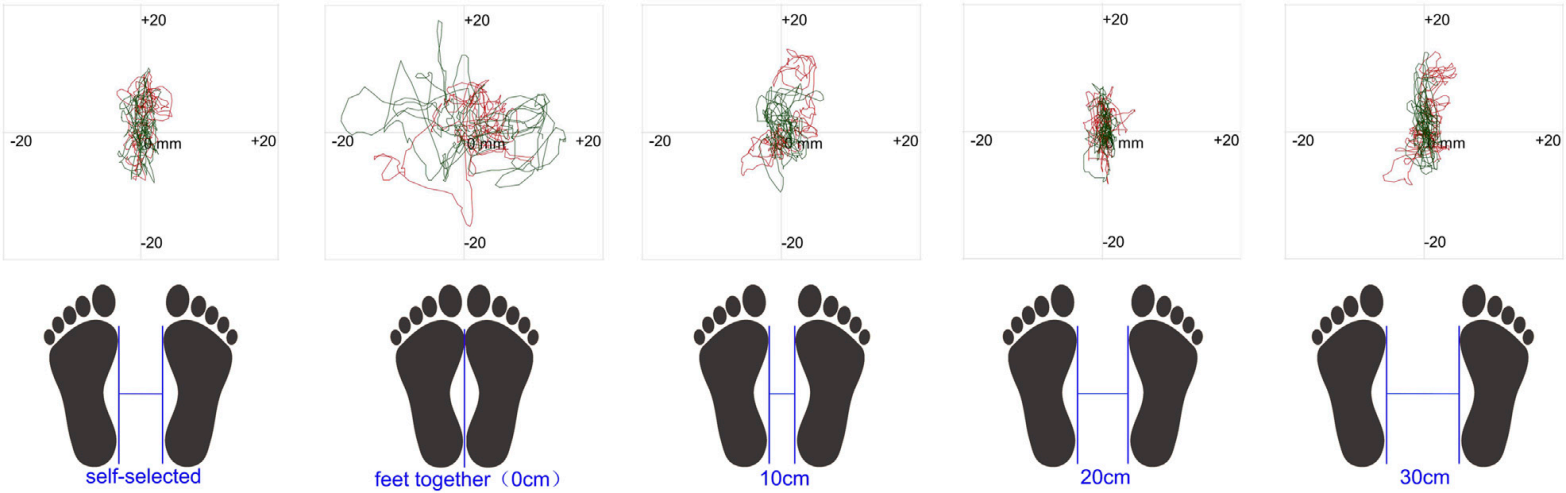
Results of a subject’s standing width program and static balance test.

The age of the 20 healthy male college students was 21.55 ± 1.39 years, with an average height of 175.50 ± 6.66 cm and a weight of 68.43 ± 12.02 kg. Figure 2 illustrates the trajectory of a subject’s swing during testing at different stance widths and during static testing. Additionally, the self-selected standing width for all subject was 16.35 ± 4.20 cm, ranging from 11 to 26 cm. In the static balance test, the rmANOVA revealed a significant main effect of standing width on all observed indicators (F > 25.097, p < 0.001, η^2^p > 0.569). Post hoc analyses indicated significant differences in certain metrics across varying stance widths between the eyes-open and eyes-closed conditions. Similarly, a significant main effect of vision was observed on all metrics (F > 37.829, p < 0.001, η^2^p > 0.666). Post hoc analyses demonstrated significant differences in some metrics between the eyes-open and eyes-closed conditions at different stance widths. Furthermore, a significant interaction between visual condition and stance width was observed for all static balance metrics (F > 23.629, p < 0.001, η^2^p > 0.554), except for the mean speed of forward and backward sway (F = 1.717, p = 0.195, η^2^p = 0.300). In the limits of stability test, a significant main effect of stance width on the magnitude of limits of stability deviation in all directions was noted (F > 12.814, p < 0.001, η^2^p > 0.403). See Tables 1, 2 for details.

**TABLE 1:** Two-way ANOVA results for static standing balance parameters.

Parameter		Main effect (stance width)			Main effect (vision)			Interaction (stance width*vision)		
		F-value	P-value	η^2^P	F-value	P-value	η^2^P	F-value	P-value	η^2^P
APAS (mm/s)	Eyes open	25.097	0.000	0.569	79.972	0.000	0.808	1.717	0.195	0.300
	Eyes closed									
MLAS (mm/)	Eyes open	134.983	0.000	0.877	84.282	0.000	0.816	95.852	0.000	0.835
	Eyes closed									
SA (mm^2^)	Eyes open	61.412	0.000	0.764	37.829	0.000	0.666	23.629	0.000	0.554
	Eyes closed									
SL (mm)	Eyes open	93.848	0.000	0.832	46.473	0.000	0.710	34.795	0.000	0.647
	Eyes closed									

**TABLE 2:** One-way ANOVA results for limits of stability (%).

Main effect	Direction			
	Front	Back	Left	Right
F-value	12.814	15.935	152.311	137.678
P-value	0.000	0.000	0.000	0.000
η^2^P	0.403	0.456	0.889	0.879

### 3.1 Effects of stance width on static balance under different visual conditions

In the eyes-closed condition, the values for each static balance index across the five stance widths were consistently higher than those in the eyes-open condition. Notably, the APAS was significantly greater in the eyes-closed condition compared to the eyes-open condition, regardless of stance width (P < 0.05). However, the MLAS showed significant differences between the eyes-closed and eyes-open conditions only at stance widths of 0 cm and 10 cm (P < 0.05), with no significant differences at other stance widths (P > 0.05). Similarly, significant differences between the eyes-closed and eyes-open conditions were observed for the SA and SL at 0 cm, 10 cm, and self-selected widths (P < 0.05), with no significant differences at other standing widths (P > 0.05).

**FIGURE 3:**
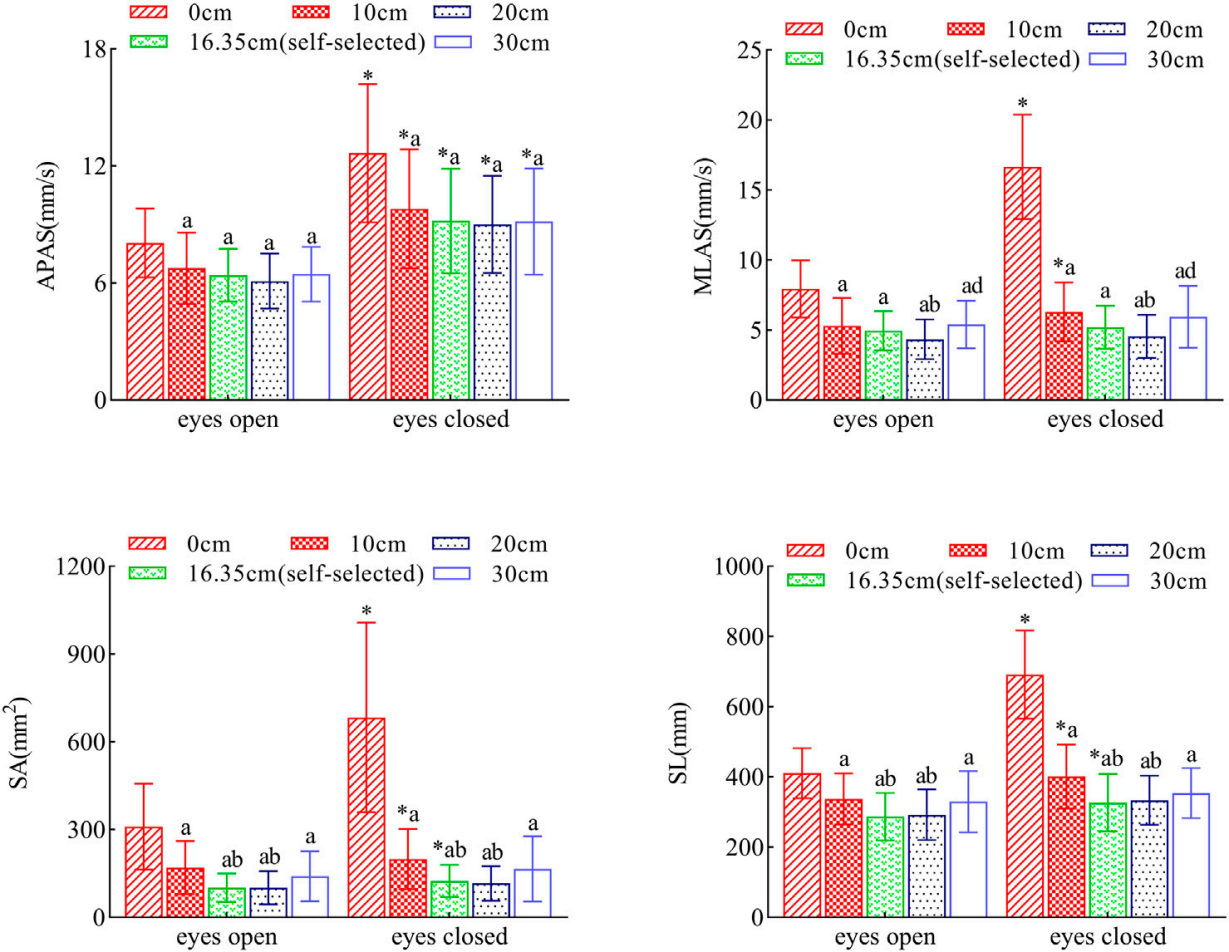
Comparative Analysis of Indicator Parameters in Static Balance Tests. Legend: *: Denotes statistically significant differences compared to the eyes-open condition (P < 0.05). (a) Denotes statistically significant differences compared to the 0 cm condition (P < 0.05). (b) Denotes statistically significant differences compared to the 10 cm condition (P < 0.05). (c) Denotes statistically significant differences compared to the self-selected width condition (P < 0.05). (d) Denotes statistically significant differences compared to the 20 cm condition (P < 0.05). Data are presented as mean ± standard deviation.

Moreover, the values of APAS, MLAS, SA, and SL exhibited a U-shaped distribution, decreasing and then increasing with increasing stance widths, reaching a minimum at the self-selected widths and at 20 cm, irrespective of whether the eyes were open or closed. It is noteworthy that when comparing the self-selected width with a stance width of 20 cm, the difference was not significant (P > 0.05). See Figure 3 for reference.

### 3.2 Effect of stance width on limits of stability with visual inputs

In the anteroposterior direction, the extent of the limits of stability increased monotonically with stance width. However, pairwise comparisons between adjacent stance widths revealed no significant differences (P > 0.05). The offset reached its maximum at a stance width of 30 cm.

In the mediolateral direction, the limits of stability also showed a monotonic increase with stance width. Specifically, pairwise comparisons indicated significant differences when the stance width increased from 0 cm to 10 cm, from 10 cm to the self-selected width, and from the self-selected width to 20 cm (P < 0.05). However, when the stance width increased from 20 cm to 30 cm, the difference in the offset magnitude in the left direction was significant (P < 0.05), whereas the difference in the right direction was not significant (P > 0.05).

**FIGURE 4:**
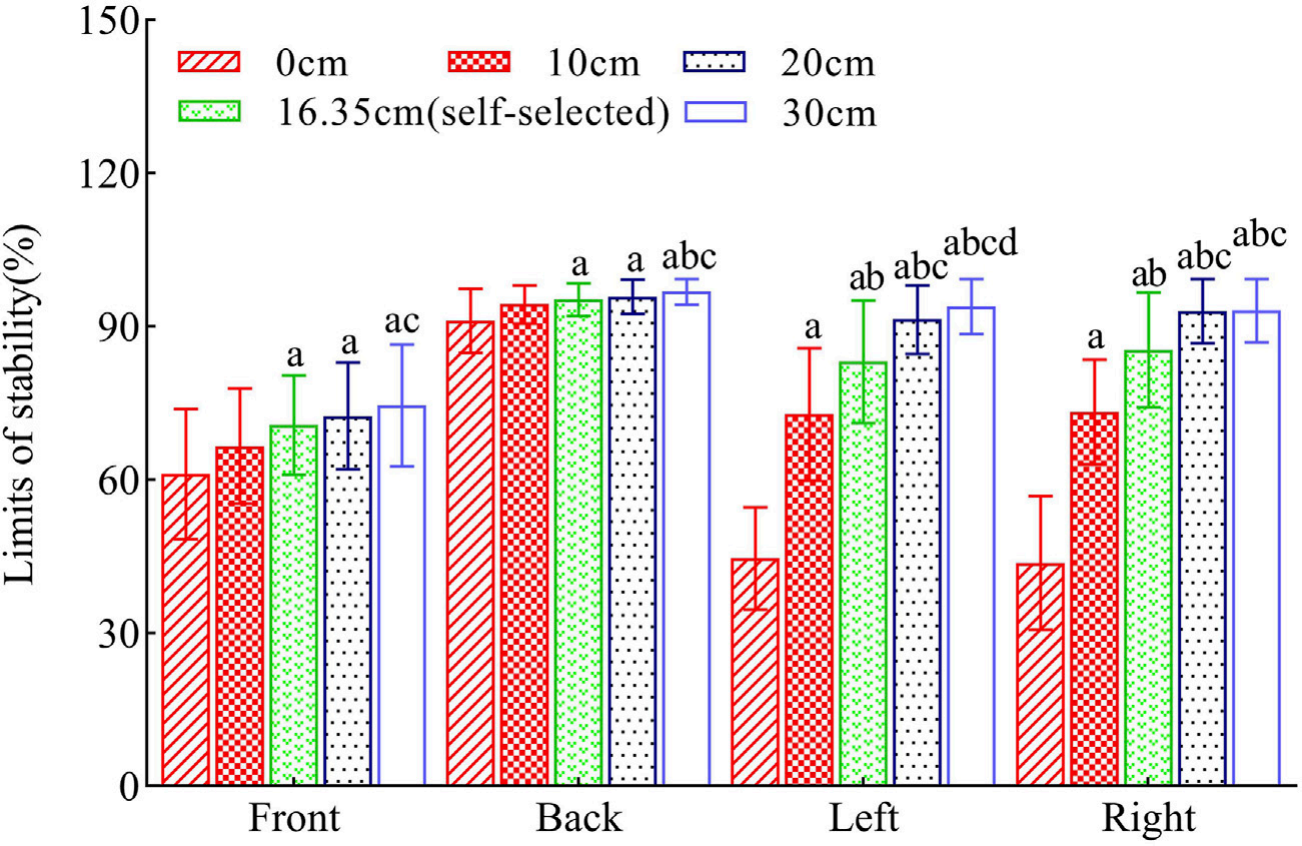
Comparative Analysis of Indicator Parameters in Limits of Stability Tests. Legend: *: Denotes statistically significant differences compared to the eyes-open condition (P < 0.05). (a) Denotes statistically significant differences compared to the 0 cm condition (P < 0.05). (b) Denotes statistically significant differences compared to the 10 cm condition (P < 0.05). (c) Denotes statistically significant differences compared to the self-selected width condition (P < 0.05). (d) Denotes statistically significant differences compared to the 20 cm condition (P < 0.05). Data are presented as mean ± standard deviation.

Notably, comparisons of offset magnitude between the self-selected and 30 cm stance widths showed significant differencesin both the anteroposterior and mediolateral directions (P < 0.05). See Figure 4 for reference.

## 4 Discussion

This study examined the effects of five different stance widths on static balance and limits of stability in healthy adults under varying visual conditions, specifically with eyes open and eyes closed. Our findings fully support the hypotheses. Specifically, static balance stability exhibited an initial increase followed by a decrease as stance width expanded, whereas the maximum excursion of the limits of stability increased monotonically. Additionally, balance stability was poorer in the eyes-closed condition compared to the eyes-open condition. Furthermore, as stance width increased, the reliance on vision for maintaining balance posture gradually diminished.

Maintaining good standing balance is essential for daily activities, preventing sports injuries, and reducing the risk of falls (Kamieniarz et al., 2018; Schedler et al., 2021). The stability of standing balance is influenced by various factors (Beelen et al., 2020; Quinlan et al., 2022), with visual input and stance width being two critical regulators (De Blasiis et al., 2023; Goodworth et al., 2014). Despite extensive research, there is no unified consensus in the current literature regarding the effects of visual input and stance width on stance balance stability (Kollegger et al., 1989). This study aims to further explore the effects of stance width on static balance and limits of stability under different visual conditions, building on previous research.

### 4.1 Analysis of static balance

Our findings indicate that both visual inputs and changes in stance width significantly affect stance balance. When examining the influence of visual inputs on static balance, we observed that across all stance widths, the APAS was significantly higher with eyes closed compared to eyes open, demonstrating a strong visual dependence that remained consistent across different stance widths. Additionally, the MLAS, SA, and SL were also greater with eyes closed, but the differences were significant only at narrower stance widths. This suggests that the visual dependence of MLAS, SA, and SL diminishes as stance width increases, aligning with the findings of Day (Day et al., 1993). This could be because the visual stabilizing effect is most pronounced when the chosen stance width correlates with balance instability (Kollegger et al., 1989). Another possibility is that visual stabilization is limited in detecting sway in the left-right direction, as proprioceptors become more effective at detecting such sway with increasing stance width, while the visual and vestibular systems are less involved (Day et al., 1993). Our study posited that visual deprivation would significantly impact body sway, especially in narrower standing postures. This hypothesis is consistent with the findings of Sarabon et al. However, their study noted varying magnitudes of change in balance parameters during postural control, likely due to the insensitivity of these parameters to visual deprivation. For example, their research showed that parameters such as frequency and capture time were insensitive to visual deprivation. They also proposed that changes in postural control parameters might result from alterations in stance width, which affect the sensory inputs used in balance control, leading to sensory reweighting (Sarabon et al., 2013).

In evaluating the impact of stance width on static balance, our study found that static balance stability initially increased and then decreased with increasing stance width. Intuitively, one might assume that a wider stance enhances static balance stability, similar to adopting a broader stance on a moving bus for stability. However, our results indicate that wider stances do not always improve balance stability, potentially due to altered coupling between ankle and hip angles (Winter et al., 1998). As stance width increases, there is heightened coupling of angular changes between the ankle and hip joints, indicating a stronger interdependence where the control of one joint may affect the other. This increased coupling can elevate the complexity of muscle coordination, requiring greater muscle control and energy expenditure to maintain balance. Consequently, the body may struggle to adapt when the stance width exceeds a certain threshold, leading to diminished postural control. This finding supports our research hypothesis that an optimal stance width exists, which minimizes postural control effort while maximizing balance stability. Our study found that subjects’ self-selected stance widths averaged 16.35 ± 4.20 cm, aligning with previous research on postural stability, which suggests that wider stances within a specific range (15–17 cm) can reduce body sway (Kirby et al., 1987). However, the comparability of results across studies may be limited by variations in the standing postures used in different investigations.

Furthermore, changes in stance width had a more pronounced effect on MLAS than APAS, likely because stance width primarily affects the frontal plane’s support area rather than the sagittal plane, a finding supported by Schmidle et al. (2022). Despite no change in sagittal plane support, APAS was still affected, contrary to Winter et al.'s findings but consistent with Hawkins (Winter et al., 1998; Hawkins and Sefton, 2011). This may be explained by the non-independence of anterior-posterior and left-right movements, where muscle activity in one plane can influence joint activity in another, related to initial sway size (Day et al., 1993). Adjustments in stance width simultaneously alter lateral stability, leading to a dynamic shift in the projection point of the center of gravity within the support plane. This shift influences the magnitude of anterior-posterior sway (Henry et al., 2001), explaining the significant impact on the APAS despite unchanged support conditions in the sagittal plane. Alternatively, it could relate to central control mechanisms, where increased stance width stabilizes overall balance, reducing swing speed, and enhancing sensory detection or motor output accuracy (Day et al., 1993). Additionally, the enhanced overall balance from increased stance width facilitates the maintenance of APAS stability. This improvement likely arises from the broader engagement of muscle groups in balance control due to the widened stance, thereby enhancing muscle proprioceptive input (Schweigart and Mergner, 2008).

### 4.2 Analysis of limits of stability

However, changes in stance width affected the limits of stability differently than static balance. Our study found that the magnitude of the limits of stability consistently increased with stance width, likely due to biomechanical shifts in the body’s center of gravity on the support surface. Increasing stance width modifies the biomechanical relationship between an individual’s center of mass and the edge of the support surface. This adjustment allows the body to accommodate a greater displacement before reaching the limits of stability (Goodworth et al., 2014; Bingham et al., 2011), thereby explaining the continuous expansion of the limits of stability parameter. A similar effect was observed in a previous study involving an older cohort (Schmidle et al., 2022). The collective evidence from our study and prior research suggests that widening stance width can positively influence the limits of stability of individuals across various age groups. Notably, the increase was more pronounced in the left-right direction than in the anterior-posterior direction, possibly because stance width changes affect the frontal plane’s support area.

Additionally, our study showed that static balance stability was optimal at a 20 cm stance width and at self-selected widths, with no significant stability difference between them. Thus, we recommend using self-selected widths to optimize static balance during posture tracing, as they require minimal effort and align with everyday stances. Conversely, the limits of stability test revealed the greatest excursion at a 30 cm stance width, significantly exceeding that at self-selected widths. Therefore, we suggest a 30 cm stance width to maximize limits of stability.

The findings of this study, particularly regarding autonomously selected stance width, significantly streamline the measurement process in postural mapping analyses. This has substantial implications, especially in contexts where physical conditions deteriorate due to aging or pathology, making it difficult to establish a fixed foot distance. Our research methodology improves the efficiency and precision of postural assessments by accommodating natural variations and individual differences in postural mapping parameters. Additionally, advocating for a wider stance width to achieve optimal limits of stability provides practical insights for maintaining balance and safety in daily activities and specific occupational settings. Furthermore, these findings enhance our understanding of multisensory integration mechanisms and hold theoretical significance by elucidating how individuals adapt their balance control strategies to varying environmental conditions.

## 5 Limitations

Several constraints are inherent in this study. Firstly, due to equipment limitations, we were unable to extend the range of standing widths tested. This limitation stems from the common use of shoulder width as a reference in human balance studies, with the average shoulder width for adult males being approximately 41 cm. Secondly, the study sample consisted exclusively of healthy males, excluding female participants, which may limit the generalizability of the findings due to potential gender-related differences in balance posture (Ozcan Kahraman et al., 2018). It is important to note that the existing literature provides inconclusive evidence regarding the impact of gender differences on balance posture. Furthermore, the study focused solely on young, healthy individuals, restricting the applicability of the findings across different age groups, as varying age cohorts may exhibit different postural responses under similar conditions (Shigaki et al., 2017).

## 6 Conclusion

In summary, visual inputs play a crucial role in stabilizing static balance across all stance widths; however, the reliance on visual cues diminishes as stance width increases in healthy adults. Our findings indicate that static balance stability initially improves and then declines with increasing stance width, while the limits of stability consistently expand. This suggests that a larger stance width is not always beneficial for static balance, whereas it is advantageous for enhancing limits of stability. Therefore, we recommend adopting a self-selected stance width to optimize static balance and a 30 cm stance width to maximize limits of stability in posture mapping.

## Data Availability

The raw data supporting the conclusions of this article will be made available by the authors, without undue reservation.
